# An Account of the Good Effects of a Decoction of Peach Leaves, in Some Affections of the Urinary Passages

**Published:** 1800

**Authors:** William Bishop

**Affiliations:** Surgeon at Maidstone, in Kent.


VII. *An Account of the good Effects of a Decoction of Peach Leaves, in ſome Affections of the urinary Paſſages. Communicated in a Letter to* Samuel Foart Simmons, *M. D. F. R. S. by Sir* William Biſhop, *Knt. Surgeon at Maidſtone, in Kent.*

ANN FULLER, a ſingle woman, aged forty-two years, has, at different times, in the courſe of the laſt five or ſix years, laboured under a ſuppreſſion of urine; and in ſome of thoſe attacks, no urine paſſed from the kidneys to the bladder for ten or twelve days each time; the catheter having been repeatedly introduced to determine this fact.

In the years 1794 and 1795, ſhe was confined to her bed ſeven months in a ſtate of great agony. The pain extended acroſs the loins, and down the courſe of the urethra, and was frequently attended with violent and long continued vomiting of blood. In the courſe of this attack, the left urethra might be felt diſtinctly in the groin, enlarged to the ſize of an hen's egg, and extremely painful when preſſed.

This

This was evidently occafioned by the preffure of calculi, which fhe afterwards voided in great number, with blood in confiderable quantities, frequently half a pint at a time, without any mixture of urine.

For her relief a variety of remedies was had recourfe to, fuch as repeated bleeding and warm bathing, faline purgatives, emetics of different kinds, camphor and opium in large dofes, uva urfi, mephitic alkaline water, &c. To the camphor, combined with opium, which brought on a copious diaphorefis, fhe was more than once indebted for a mitigation of her painful fymptoms. The mephitic alkaline water was tried repeatedly, in different forms, plain, and with additions, cold and warmed, but it conftantly occafioned pain of the ftomach and vomiting.

At length, the haematuria continuing, accompanied with a good deal of pain, and every remedy that had been adminiftered, having failed to relieve her effectually, Mr. Gabriel Allen, my affiftant, fuggefted to me a trial of a decoction of peach leaves, from which he had occafionally feen good effects in cafes of nephritis. He was firft led, it feems, to the ufe of this remedy by a perfon, not of the medical profeffion, who was much reforted to by by patients labouring under complaints of this kind, and who made a very fuccefsful ufe in fuch cafes, of an electuary, compofed of honey, and peach leaves dried and powdered; together with a decoction or infufion of the leaves.

After having feen fo many other remedies fail in this cafe, I was anxious to try the effect of this new medicine. I fay *new*, for although different writers on the materia medica, mention the anthelmintic properties of the leaves, and likewife of the flowers, of the peach tree, I do not find that any of them have noticed their effects in affections of the urinary paffages.

A decoction was accordingly prepared, by boiling an ounce of dried leaves of the peach tree, (*Amygdalus Perfica* Linn.) in a quart of water, till it was reduced to a pint and a half. Of the ftrained liquor fhe took a pint daily, and at the end of thirty hours after fhe began the ufe of this remedy, fhe voided clear natural urine, and in a few days recovered.

From that time fhe has conftantly kept by her a quantity of the dried leaves, and on the leaft return of the fymptoms has had recourfe to the decoction again. Since that period, fhe

ſhe has had ſeveral ſlight returns of gravel, and has even paſſed ſome ſmall calculi, but ſhe has had no return of the haematuria. Her preſent comfortable ſtate of health ſhe attributes to the uſe of the decoction of peach leaves; at any rate, it ſeems to be deſerving of a trial in ſimilar complaints. I have tried it in a variety of inſtances beſides the one which is more particularly the ſubject of the preſent letter, and I am deceived if it is not a medicine of conſiderable efficacy in complaints of this kind. Upon theſe grounds it is that I have ventured to recommend it to your notice.

*Maidſtone, Dec.* 12, 1797.

VIII. *A*

